# The experiences of students with intellectual and developmental disabilities, parents, and teachers regarding health self-advocacy program with school-home connection: a qualitative study

**DOI:** 10.3389/fpsyt.2023.1273324

**Published:** 2023-10-24

**Authors:** Suk-Hyang Lee, Ha-Nul Kang, Soyeon Kim

**Affiliations:** ^1^Department of Special Education, Ewha Womans University, Seoul, Republic of Korea; ^2^Department of Special Education, The Graduate School of Ewha Womans University, Seoul, Republic of Korea

**Keywords:** students with intellectual and developmental disabilities, health self-advocacy, stigma, self-determination, qualitative study

## Abstract

**Background:**

Despite the importance of health as a significant indicator of quality of life, individuals with intellectual and developmental disabilities (IDD) often face low expectations, stigma, and insufficient opportunities in health care and education. In response, we developed a health self-advocacy program with a school-home connection for students with IDD to promote self-directed health care and verified its effectiveness by implementing the program for students with IDD.

**Objective:**

This study aimed to explore participants’ program experiences and support needs to reduce the stigma surrounding individuals with IDD and provide implications for enhancing health self-advocacy skills.

**Methods:**

Individual and focus group interviews were conducted with 14 students, six parents, and four teachers who participated in the program. The collected data were analyzed using the constant comparative method.

**Results:**

The following five main themes emerged: (a) the gap between perception and practice in health care; (b) advantages and influencing factors of the program; (c) challenges in program implementation; (d) outcomes of program implementation; and (e) support needs for promoting health self-advocacy.

**Conclusion:**

Based on these findings, implications are provided and discussed to reduce the stigma surrounding individuals with IDD and enhance health self-advocacy.

## 1. Introduction

Health is a critical indicator of the quality of life (1), and a healthy life is a significant value and human right for all individuals, including those with disabilities. Article 25 of the United Nations “Convention on the Rights of Persons with Disabilities” guarantees the right to non-discriminatory access to health care and equal access to medical services for people with disabilities (2). Especially during the unprecedented crisis caused by the COVID-19 pandemic, international organizations have emphasized the non-discrimination of people with disabilities to ensure education and the right to health (3–5) and have issued recommendations to prevent medical inequalities (6). Accordingly, in response to the heightened attention paid to individual health and quality of life owing to the global infectious disease outbreak, South Korea has established institutional support measures to protect the health rights of people with disabilities, including the development of guidelines for COVID-19 response for people with disabilities (7).

However, despite this institutional support, people with disabilities have been unable to escape the issues of health support and health care service inequalities caused by COVID-19. The impact of COVID-19 is more severe for those in lower socioeconomic positions or marginalized groups (8). Residents in impoverished areas or ethnic minorities have lower vaccination rates, and women experience significant anxiety owing to COVID-19, highlighting the exacerbation of existing risk factors in times of crisis (9, 10). Among these groups, people with intellectual and developmental disabilities (IDD) have experienced the most significant negative impact and medical inequalities resulting from COVID-19. They face a higher risk of exposure to COVID-19, have higher infection and mortality rates due to underlying conditions, and experience increased anxiety and depression as well as challenging and aggressive behaviors (11–16). Furthermore, they have difficulties accessing information related to infectious diseases or medical support (14, 16–18). Even in South Korea, individuals with IDD face significant constraints in accessing health care services, which can be attributed to a lack of understanding of their disabilities among medical professionals and communication difficulties. According to the Survey on the Status of Persons with Disabilities (19), the rate of health checkups among individuals with disabilities is lower than among individuals without disabilities, and this constraint is more pronounced among individuals with IDD. Furthermore, the prevalence of chronic conditions among individuals with IDD in South Korea was 54%, which was higher than that of individuals without disabilities and other disability types. Approximately one-third of individuals with IDD reported being unable to visit a hospital when needed within the past year. Communication difficulties have been reported as the primary factor related to these issues.

The characteristics of individuals with IDD, such as cognitive limitations and communication difficulties, can become factors in the stigma surrounding individuals with IDD, leading to health care and medical inequalities (19–23). This demonstrates the need to strengthen the capacity of individuals with IDD to manage their health beyond institutional support to guarantee health rights. The importance of self-directed health care is also emphasized in the redefinition of health as the ability to adapt to and manage individual challenges faced in physical, social, and psychological aspects (24). In the same context, self-determination can lead to the active participation of individuals with IDD in medical decision-making processes (25–27), and health self-advocacy, which involves actively expressing one’s opinions and concerns about health and advocating for oneself in health care and medical situations (28, 29).

Health self-advocacy for individuals with IDD is critical for the following reasons: First, health, which is an essential indicator of the quality of life, affects the lives of individuals with IDD and their families. For instance, the health status of adults with IDD significantly impacts their life satisfaction more than their economic characteristics (30). The more severe the health problems of individuals with IDD, the more negative the impact on their cognitive functioning and daily living performance (31). Furthermore, as the health status of adults with IDD becomes severe, the burden of care on parents also increases (32), indicating that personal health affects individuals themselves and the quality of life for their families.

Second, the health of individuals with IDD is related to their transition to adulthood. The physical and mental health status of individuals with IDD was found to be associated with employment, one of the outcomes of the transition to adulthood (33), and the need for self-management and personal hygiene was highlighted to maintain employment for individuals with IDD (34).

Third, individuals with IDD often face limitations in basic health knowledge and access to information, making it difficult for them to adequately express their illness or pain, which can ultimately lead to secondary health issues (25, 35, 36). Furthermore, many cases also exist where passive health care by family members or caregivers, rather than individuals with IDD themselves, occur because of communication limitations and low expectations toward them (37). In South Korea, individuals with IDD made primary decisions in only 28.6% of cases. In comparison, parents made decisions in 50.4% of the cases (38), indicating the need to enhance health care capacity to enable individuals with IDD to take a more proactive approach to managing their health.

Fourth, despite the lack of expectations and the resulting lack of health care opportunities for individuals with IDD, direct intervention studies that can enhance the health care capacity of individuals with IDD are lacking (39, 40). Various educational programs and curricula, including health self-advocacy training, have been developed internationally to enhance the self-directed health care of individuals with disabilities, and their effectiveness has been investigated. For example, health self-advocacy training affects knowledge about the health rights of people with intellectual disability and health self-advocacy skills (41), and school-based health advocacy interventions were found to increase health care activities among adolescents with intellectual disability (42). The Ask Health Diary, developed for communication and self-advocacy related to health, was reported to help young people with intellectual disability advocate for their health and promote self-determination (43). In South Korea, a health education program for young adults with IDD was effective in improving participants’ health knowledge and health-promoting behaviors (44), and a health education program for mothers of children with IDD had a positive influence on their children’s health-promoting behaviors (45). However, there have been few studies on health-related issues in individuals with IDD or on their parents in South Korea, and most health-related intervention studies targeting individuals with IDD have focused primarily on exercise programs for physical and fitness improvement (39). Moreover, consistent education and support between schools and homes are necessary to provide comprehensive education and support for the health care and education of students with IDD (37, 46), and health self-advocacy programs can be an opportunity to enhance cooperation between teachers and parents (43).

Considering these points, we developed a health self-advocacy program with a school-home connection to reduce the discrepancy between demands in the field regarding the health of individuals with IDD and research (47). Furthermore, we implemented the program for high school students with IDD and investigated its effects by analyzing the data collected before and after the program. The results of the analysis revealed that the program positively impacted health-related knowledge, behaviors, goal achievement, and self-determination as perceived by students with IDD. However, there were limitations in demonstrating the fundamental changes and maintaining lifestyle habits related to health care among the research participants. Therefore, beyond the quantitative analysis of intervention research, the purpose of this study was to explore the experiences and support needs of students with IDD, their parents, and teachers who participated in the program, and to investigate their perceptions of health and health care along with changes and maintenance in self-directed health care habits. Furthermore, based on the findings, we aim to propose implications for breaking the stigma associated with low expectations of students with IDD and reducing inequalities in health care and support, as well as implications for enhancing their health self-advocacy.

## 2. Materials and methods

### 2.1. Participants

The participants comprised 14 high school students with IDD, six parents who volunteered to participate in interviews, and four special education teachers. All participants attended our previous intervention study (47) and were selected based on the following inclusion criteria: (a) high school students with IDD who could understand simple directions or questions and answer questions regarding their experiences with or without verbal prompts or visual aids (e.g., simplified text or pictures) and (b) students with IDD, their parents, and teachers who did not have any previous experience of participating in interventions to enhance health self-advocacy and agreed to participate in the intervention. As depicted in Table 1, among the 14 students, nine (64.3%) were in the second grade, four (28.6%) in the third grade, and one (7.1%) in the first grade. Thus, second-year students comprised the highest number of students. Regarding disability types, nine students (64.3%) had intellectual disability (ID), and five students (36.7%) had autism spectrum disorders (ASD), making intellectual disability the most common type. Six of the students needed communication support during the interview (e.g., verbal prompts or visual aids) to clarify the meaning of the interview questions, and two of them had specific health issues such as diabetes (S5) and severe obesity (S2). All parents of students with IDD were mothers, with an average age of 48.7 years. The teachers included one male (25%) and three females (75%) with an average age of 38.8 years, and their teaching experience varied from 3 to 25 years (see Table 2).

**Table 1:** Background information of students and parents.

Students					Parents		
Number	Age	Grade	Sex	Disability	Number	Age	Sex
S1	18	2	Male	ASD	P1	48	Female
S2	17	2	Male	ASD	·	·	·
S3	18	2	Male	ID	P3	49	Female
S4	17	2	Male	ID	–	–	–
S5	17	2	Male	ID	–	–	–
S6	18	3	Male	ID	–	–	–
S7	18	3	Male	ID	P7	47	Female
S8	18	3	Male	ID	–	–	–
S9	18	3	Male	ASD	–	–	–
S10	16	1	Male	ID	P10	52	Female
S11	18	2	Male	ID	–	–	–
S12	18	2	Female	ID	–	–	–
S13	17	2	Male	ASD	P13	49	Female
S14	17	2	Male	ASD	P14	47	Female

**Table 2:** Background information of teachers.

Number	Age	Sex	Years of experience*
T1	38	Female	17
T2	32	Female	6
T3	28	Male	3
T4	57	Female	25

*Total years of experience as a special education teacher.

### 2.2. Intervention

All participants attended the health self-advocacy program with a school-home connection that we developed. The program consisted of 15 sessions, including orientation, basic and advanced lessons in six health care domains (i.e., hygiene, exercise, eating/nutrition, stress, danger/safety, and hospital/medicine), a review session, and a completion ceremony. The program was implemented once a week for 50 min in a special education classroom over 5 months with 14 students with IDD to enhance their health self-advocacy. During each basic lesson on health domains, students with IDD assessed their health conditions and health care behaviors related to the domain, set a goal, and developed a plan to meet goals based on the results of the self-assessment using a Collaboration-based Instruction Model for Self-Determination (CIMSD) (48) and goal attainment scaling (GAS) (49) to help them develop and monitor their goals and plans for 2 weeks at school and home. During each advanced lesson on health domains, the students applied what they had learned through each basic lesson to solve health and health care problems. Parents and teachers supported the students in taking action to reach the goal and monitored their performances at home and school by encouraging and reminding them to work on their goals using self-checklist sheets designed to check their daily performance.

### 2.3. Data collection

We developed an interview guide for students, parents, and teachers to explore their perceptions of health self-advocacy, participation experiences, and support needs regarding the program, including the following common questions: What do you think a healthy person and health self-advocacy mean? What was the most helpful aspect of participating in this program? What were the most challenging aspects of the program? What were the most significant changes you experienced in the program? Are you still making an effort to manage your health? What kind of support do you require to enhance your self-advocacy for health?

This study employed different interview methods, considering the characteristics and convenience of the teachers, parents, and students. First, a focus group interview was conducted with teachers within 2 weeks of the program’s completion through video conferencing. The first author conducted teacher focus group and parent interviews. Second, interviews with parents were conducted within 3 weeks of the program’s completion via video conferencing or phone, depending on the parents’ convenience. During the parent interviews, the first author provided an overview of the program’s content and structure to remind them, along with the individual goals set by their children. Finally, individual interviews were conducted with students to examine the maintenance of program effects one and a half months after the program’s completion. The coauthors conducted individual student interviews through video conferencing. Visual aids were provided including simplified text and illustrations, and screen sharing was used as a reference during the interviews. All interviews were recorded with the participants’ consent; the length of the focus group interview with the teachers was about 71 min, and the average length and range of the parent and student interviews constituted approximately 29 min (28–57 min) and 26 min (12–22 min), respectively. The interview recordings were transcribed using the free AI speech-to-text software *clovanote*^1^. The coauthors then listened to the interview audio files, verified the transcription’s reliability, and corrected errors.

### 2.4. Data analyses

The transcripts were analyzed using the constant comparative method (50). Identical copies of the transcripts were independently read to code the data, establish operational definitions, and perform open coding. Through discussions centered on each aspect of open coding, we organized the codes and their interpretations to develop the initial codebook. Using this codebook, we independently applied coding to the remaining transcripts. Subsequently, we conducted an open discussion to determine whether new codes were necessary or whether any modifications, integrations, or additions were required to update the codebook. We thoroughly analyzed each interview transcript and identified five primary themes. Additionally, efforts were made to avoid the potential influence of researchers’ subjective perspectives on the analysis process and enhance the trustworthiness and validity of the research (51) as follows: First, all authors shared opinions and conducted the analysis through collaborative discussions, thereby ensuring the credibility of the analysis through the process of triangulation among analysts. Second, we provided the results of this study to parents and teachers for member checks so that they could review and confirm the appropriateness of the results, including themes and citations. Based on participant feedback, no additional modifications were requested regarding the research findings.

### 2.5. Researcher positionality

The first author has worked as a special education teacher at a special school and a regular school and has conducted research on health inequity from the perspective of self-determination in people with IDD. The second and third authors are both doctoral students majoring in special education and have worked as special education teachers. Based on our teaching experiences and academic backgrounds, we considered this topic to be important for students with IDD, given that students with IDD are more likely to be excluded from health education and to depend on others without the motivation to take care of themselves. These concerns and our backgrounds as researchers and practitioners in the field of special education guided us to interview all the participants with empathy and to recognize their needs beyond journal articles from an insider’s perspective. However, we interpreted the participants’ experiences and needs from an “in-betweeners” perspective, which identified us as being neither insiders nor outsiders (52).

## 3. Results

**Figure 1:**
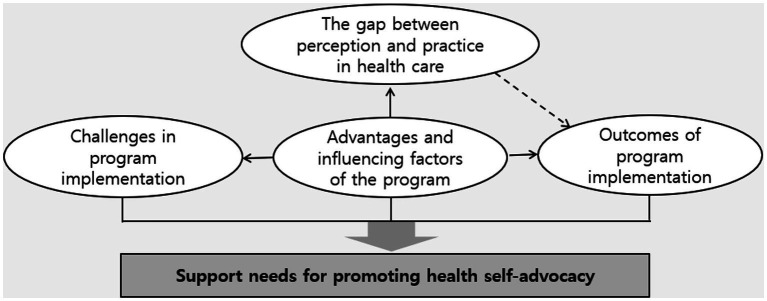
Conceptual relationship between the main theme.

Analysis of the interview data resulted in five main themes and 12 subthemes, as presented in Table 3. Furthermore, a conceptual relationship between the main themes was observed, as depicted in Figure 1.

**Table 3:** Derived themes and key content.

Main themes	Sub-themes	Key content
1. The gap between perception and practice in health care	1.1 The importance of health self-advocacy	Self-directed health care
		Prevention and promotion of health issues
		Positive transition outcomes into adulthood
	1.2 Challenges in health care	Insufficient regular exercise
		Difficulties in dietary habits and nutrition management
		Challenges in weight control
		Lack of personal hygiene habits
2. Advantages and influencing factors of the program	2.1 Advantages of program content and structure	Comprehensive coverage of various aspects of health management
		Harmonization of knowledge and application by basic and advanced sessions
		Establishing health care goals and action plans
	2.2 Advantages of program implementation	School-home connection
		Various supports for planning and monitoring
		Using various interesting teaching materials
	2.3 influencing Factors of the Program	More favorable acceptance of instructions from teachers compared to parents
		Voluntary effort and determination
		Motivation and awareness leading to practical actions
3. Challenges in program implementation	3.1 Challenges of teachers in program implementation	Insufficient class time and application duration
		Limitations of school-home connection
	3.2 Challenges of students and parents in program participation and support	Challenges in monitoring and implementing action plans
		Difficulties in consistent practice
		Limitations of home support
4. Outcomes of program implementation	4.1 Changes and outcomes regarding students	Changes in perception regarding health and health self-advocacy
		Changes in health care behaviors across different areas
		Maintenance of behavior changes and willingness to health care
		Generalization of learning experiences through the program
	4.2 Changes and outcomes regarding teachers and parents	Changes in perception of health self-advocacy
		Awareness of the need for teaching health self-advocacy and willingness to teach
		Generalization of goal setting, planning, and support-related practices
		Understanding of students’ present performance levels
5. Support needs for promoting health self-advocacy	5.1 Advanced health care education and professional support	Specialized education in various health care areas
		Stress management and emotional support
		Expert support and counseling
		Expansion of health education content
	5.2 Activation of health self-advocacy programs	Inclusion of regular classes and confirmation of maintenance and generalization effects
		Early and long-term education
	5.3 Enhancement of teachers’ and parents’ capacities regarding health self-advocacy	Strengthening teachers’ capacities for teaching health self-advocacy
		Enhancing parents’ capacities for supporting health self-advocacy

As demonstrated in Figure 1, the participants were aware of the importance of health care and health self-advocacy. However, there was a gap between their perceptions and actual health care practices. Nevertheless, participating in a health self-advocacy program with a school-home connection resulted in positive changes and outcomes for students, parents, and teachers, as indicated by the advantages and influencing factors of the program. Despite these positive results, the participants reported experiencing various challenges during the program’s implementation. Considering the features and outcomes of the program, along with these challenges, support is required to enhance health self-advocacy.

### 3.1. The gap between perception and practice in health care

#### 3.1.1. The importance of health self-advocacy

Most students with IDD were aware of the importance of self-directed health care. They recognized the need to take care of their health and emphasized the importance of health care in preventing personal health issues, enhancing immunity, and reducing stress.

*I do it myself (Researcher: Why do you do it yourself?) Because I have to take care of my health (S6).*

In contrast, parents and teachers considered health self-advocacy to be important for positive transition outcomes into adulthood. They mentioned the importance of personal hygiene and health care in becoming independent members of society after graduating from high school without relying on parental assistance.

*When they graduate and live as adults, they can live as healthier members of society, and enhancing health self-advocacy will be helpful to them (T2).*

*As S13 enters the third year of high school and will enter society in the year after next, when they go out into society and engage in activities, hygiene issues are really important (P13).*

#### 3.1.2. Challenges in health care

Although most participants recognized the importance of health self-advocacy, a gap existed between perception and practice because of factors such as a lack of health care habits. Notably, a lack of regular exercise was identified as a significant challenge. They reported being unable to exercise for personal health reasons (e.g., atopy and heart disease) and external conditions, including COVID-19.

*S13 likes to stay at home, and I also started to prefer staying at home because of COVID-19. I used to exercise a lot before (P13).*

In addition to a lack of exercise, difficulties in managing inappropriate eating habits such as picky eating, late-night snacking, overeating, and challenges in maintaining weight have been reported. A general difficulty in forming proper habits related to exercise, diet, and personal hygiene was observed, such as brushing teeth, owing to laziness or forgetfulness. A small number of participants mentioned the difficulty of communicating pain to health professionals as a barrier to health care.

*It is hard for them to eat at night, so I told them to eat less, but they could not follow that. It would be better if they ate less and reduced mealtime, but they eat too much (P10).*

### 3.2. Advantages and influencing factors of the program

#### 3.2.1. Advantages of program content and structure

In terms of the advantages of the program’s content and structure, the participants highlighted that the program covered a comprehensive range of health care, not only physical health aspects such as hygiene and dietary management typically addressed in traditional health education but also encompassed areas such as stress management, prevention of risks such as smartphone addiction, and overall mental health.

*This program covered how to manage stress, how much we use our smartphones, and the risk factors. It comprehensively covered mental and physical health aspects, so it was great (T4).*

Furthermore, dividing the program into basic and advanced levels for each health care domain allowed participants to receive basic information about health care in each domain and then apply it specifically. The participants mentioned that this approach helped them form their health care habits. In particular, setting individual goals and planning for their goals in health care enabled students to actively practice health care.

*It was helpful for them to set their own goals. Instead of me telling them what they should do, allowing them to set their own goals and practice self-management was really helpful for them throughout the program (T1).*

#### 3.2.2. Advantages of program implementation

In addition to the program’s content and structure, advantages related to its implementation have been reported. First, parents who participated in the study reported that it was challenging for their children to consistently implement health care practices at home. However, through school-home connections, parents were able to support their children’s health care goals at home by helping them implement action plans.

*Honestly, it was not easy to set and maintain goals together. However, because the school kept providing support and teachers kept talking about it, that alone was a tremendous help for us (P3).*

During the school-home connection, various supports were provided to monitor whether students kept working on their health care goals. This support included visual aids such as self-checklists and assistance from peers, parents, and teachers (e.g., providing language stimulation, capturing proof shots or videos of students’ exercise routines), and sharing proof shots, which are photos of students’ actions and practices to reach their health care goals, in a group chat with other peers as proof of their practices. Sharing these photos of students’ practice in the group chat allowed self-checking and provided a channel for positive interactions with peers.

*The goal check sheet was helpful. It allowed me to check if I was doing well, which helped me do better (S7).*

*Sometimes I forget, so my sister helps me (S4).*

Teachers also appreciated the use of various multimedia materials suitable for engaging students during class. Additionally, conducting classes with small groups of students of different ages provided positive role models for students.

*In addition, it was good that we used different and innovative materials for the activities. The kids could concentrate better on those (T2).*

#### 3.2.3. Influencing factors of the program

Regarding the factors influencing the program’s implementation, some students were more likely to listen to their teachers’ recommendations than their parents. They were motivated to practice health care at home based on what they had learned in school from their teachers.

*My child only listens to about 30% of what I say, but my child does it very well when the teacher says it. I relied on the teacher a lot (P7).*

Furthermore, students’ voluntary efforts and willingness to check goals and implement actions, even when lacking support at home, were mentioned as positive influences on the program’s implementation. Conversely, the lack of motivation to exercise diligently at school or to maintain regular dietary habits at home negatively affected the program’s implementation. Additionally, awareness of personal health risks identified through health checkups and motivation and encouragement from teachers and peers were also reported as influencing factors in the program.

### 3.3. Challenges in program implementation

#### 3.3.1. Challenges of teachers in program implementation

The teachers expressed satisfaction with the program’s content. However, they mentioned the challenges of covering a significant amount of content in a single session owing to limited class and application time. Moreover, they reported challenges regarding the extent of parental support, which varied depending on the characteristics of each home. Teachers also had trouble actively requesting support from parents or verifying the level of support provided at home.

*What was a little regrettable is that the time was too tight despite the great content. The content was not difficult, and I felt the students could digest it sufficiently. However, we had to rush through it, which was a bit disappointing (T1)*

#### 3.3.2. Challenges of students and parents in program participation and support

In addition to the challenges in program implementation mentioned by the teachers, difficulties with student participation and parental support have been reported. Both the students and parents mentioned challenges in self-monitoring their planned actions. Students took a lot of time to keep up with daily self-monitoring sheets and, in some cases, lost their checklists, leading to delayed or incomplete checks. While sharing proof shots in the group chat helped some students stay motivated to take action to reach their goals, it also felt burdensome to others as they felt the need to impress their friends. Moreover, some students faced conflicts with their parents during the goal-setting process because of their different ideas about the goals.

*It was challenging to remember and check every day. I was confused about when and how many times I did things. So I just checked them off hastily (S4).*

*In one student’s case, from the beginning, the goal set by the child did not sit well with the father. So, there was a conflict with his father. His father did not like the goals the child set and asked him to use something different, like eating tomatoes, even though he did not like them (T1).*

Furthermore, both students and parents faced difficulties maintaining consistent health care practices. Although they understood the importance of health care, there was a gap between their awareness and actual practice. For instance, although reducing smartphone use was beneficial, some found it challenging to practice. While setting goals was beneficial, they struggled to maintain consistency because of their existing habits. Other difficulties included health issues and physical limitations affecting regular exercise and dietary management.

*What was difficult was being consistent. I found it hard to consistently do things that I did not usually do. Establishing these habits was challenging (S11).*

In addition, some parents, especially working mothers, faced difficulties in providing consistent support for their children’s health care due to limited time and energy.

*Sometimes, I have to work until midnight. As a result, I could not provide proper support for the checklist, which happened too often. I felt bad about it and sometimes ended up asking the teachers for help (P7).*

### 3.4. Outcomes of program implementation

#### 3.4.1. Changes and outcomes regarding students

First, notable student changes resulting from program participation were related to students’ perceptions and willingness regarding health and health self-advocacy. After participating in the program, the students recognized that a healthy person possesses both physical and mental well-being. The participants acknowledged that engaging in activities related to personal hygiene, exercise, diet management, and stress management classified them as healthy. Moreover, they understood the significance of willpower in health care, emphasizing that exercising is essential for maintaining good health, even when it is inconvenient.

*First, to be healthy, you need a strong body, then you need to eat well and play well and then, always smile, and a person who smiles seems to be a healthier person (S10).*

*A healthy person takes good care of their body. Even when they feel lazy, they still think that they have to do it, so they do it even though it is bothersome. I think a person like that is healthy (S5).*

Furthermore, students developed a willingness to take responsibility for their health care. Both students and parents reported that the health self-advocacy program with the school-home connection not only helped them realize the importance of self-directed health care but also encouraged them to pursue the health goals they set for themselves in their daily lives.

*What I found most significant was their efforts to take care of themselves and self-regulate. They understand why it is good in class and set their own goals, so they plan and do something like self-management or self-regulation (T2).*

*Now I think I have to take care of my health myself. Otherwise, I feel tired and unhappy a lot, so I think I have to take care of my health by myself (S10).*

Second, voluntary health care behaviors in various health domains followed awareness and willingness to prioritize health. Notably, students who initially struggled with goal setting could set concrete, achievable goals for each health care domain and make efforts to implement them as the program progressed.

*The good thing was that when students set challenging goals, we wondered if they could do them. Recently, the children have been trying to find things that they can realistically practice (T4).*

These positive changes have led to the development of healthy habits in various areas. For instance, students consistently pursued their exercise goals, such as using a treadmill, running on the school playground, biking, and performing push-ups, without needing reminders from others. Some students used apps such as CashWalk to check their daily walking goals. Regarding personal hygiene, the students actively engaged in practices such as brushing their teeth, washing their faces, and showering more consciously than before. Additionally, some students looked in the mirror and tried to tidy themselves up before school. Parents reported that they had less need to nag their children about their personal hygiene habits.

*I used to not shave much before. But now, through this program, I have formed a habit of shaving (S11).*

*When they come back from lunch, instead of just sitting and using their phones, they brush their teeth (T4).*

Furthermore, positive changes in students’ dietary habits were evident. Students reduced their consumption of carbonated drinks and instant foods like ramen. They tried to include more vegetables in their meals and cut back on nighttime snacks and sodium-rich soups. Many students began eating breakfast regularly or paid more attention to having a balanced meal with side dishes.

*I used to eat a lot of fast food, like ramen and hamburgers, but now not so much (S4).*

*I used to drink a lot of carbonated drinks, but now when I crave them, I drink milk instead and endure it. Also, I used to eat a lot of greasy food, but now I changed my diet to focus on vegetable side dishes (S11).*

Moreover, students demonstrated proactive stress management by engaging in activities they enjoyed to relieve stress, such as listening to music, exercising, and playing games. They also reported reduced smartphone usage by finding alternative activities to replace their excessive gaming time, as indicated by their self-assessment. In addition to stress management, the students acquired basic first-aid skills, demonstrating their ability to handle simple emergencies. Regarding medication, students took a more proactive approach to medication intake, planned when to take their medications, and adhered to their plans rather than making excuses or forgetting to take them.

*When I feel stressed while doing something, it helps me to listen to music or exercise (S5).*

*Some students talked about reducing their phone usage and replacing it with other activities, such as exercising together during lunchtime. Even now, they continue these activities (T1).*

Third, positive behavioral changes persisted after the program’s completion and reflected students’ willingness to continue their health care efforts in the future. Many students expressed their intention to maintain their health habits, such as exercise, dietary practices, and stress management, over the long term.

*Although the exercise program ended a while ago, some students put exercise in place of the time they used to spend on their phones. They go outside together during lunchtime to exercise. They still continue this practice (T4).*

Finally, another change observed in the students was related to the generalization of the program’s effects. They applied checklists for planning and implementation in classroom situations, such as checking the weather and paying attention to their clothes while going out. Most importantly, there was a positive change in the classroom atmosphere, with students engaging in activities, such as brushing their teeth and exercising together during lunchtime. Some students experienced increased self-esteem and a sense of accomplishment when achieving their goals.

*Students used to sit and look at their phones or stay still during lunchtime, but now they all go to the playground and play, enriching their school lives. It was not only about physical health but also about significant changes in their school lives (T4).*

*It is hard for our kids to boost their self-esteem, no matter where they go. But now, they gain confidence through experiences, achieve their goals, and feel proud. This boosts their self-esteem. Even in this aspect, I feel happy to see my child achieve their goals and experience the joy of accomplishment (P14).*

#### 3.4.2. Changes and outcomes regarding teachers and parents

First, the most significant outcome observed in the teachers and parents who participated in the program was a change in their perception of health self-advocacy. Before the program, participants regarded health self-advocacy as proactive health care or simple self-management. They also considered it a challenging topic to teach during class because of its difficulty for the students. However, after the program, their concept of health self-advocacy became clearer by considering it as implementing specific goals and plans for health care and bringing about a shift in their expectations of students.

*Improving health self-advocacy capacity is an area that is closely related to ours, but even I found it somewhat bothersome and difficult to teach. Now it would be great to teach similarly in the future (T4).*

*I used to think that health self-advocacy was about living in a healthy direction, but through this program, I can think more deeply about how to practice health care (T2).*

Before students participated in the program, parents had varied understandings of health self-advocacy, ranging from not knowing about it to understanding it as self-directed health care and acquiring the necessary knowledge for health. However, as they observed their children’s proof shots regarding health care practice to reach their goal in the group chat, they recognized that health self-advocacy enhances their children’s capacity for health care.

*I did not really understand what health self-advocacy meant. However, now I have begun to perceive it as strengthening their capabilities, as I saw the proof in the group chat (P14).*

Second, teachers reported experiencing positive effects on students and parents, which made them realize the program’s significance and increased their willingness to do more. Some teachers from the same school did not participate in the program but displayed interest after seeing changes in the students. Parents also mentioned that they were more conscious of guiding their children in health care through school collaborations.

*After that, our lunchtime routine changed. The teacher next door asked me how she could join this program. She mentioned that it seemed really interesting based on how much the students have changed due to this program (T4).*

Third, the teachers who participated in the program generalized the experiences gained from supporting students’ goal-setting and taking action to reach goals in other areas. For example, they planned to integrate health self-advocacy with the Individualized Education Program (IEP) and implement it in coordination with other subjects such as public transportation and school life. Parents also discovered that, by empowering their children to set goals, they could manage their health and reduce the pressure they felt about their children’s care.

*S1 was gaining weight and struggling with diet management at that time. However, through this program, I see that he will manage his health if S1 has goals and recognizes them (P1).*

Finally, the program provided teachers with more insight into students’ performance through self-assessment and self-diagnosis in health areas during class time, proof shots in group chats, and checklists at home. These insights will allow teachers to better understand their students and consider directions for future instruction.

### 3.5. Support needs for promoting health self-advocacy

Participants reported the following support requests regarding future health self-management programs: First, they emphasized the need for advanced health care education and specialized support. This included in-depth education in various health domains (e.g., specific nutrition counseling and more detailed stress management techniques); emotional support for families, including stress management for children; and demands for professional assistance and counseling. Additionally, they suggested expanding health education to cover the self-expression of pain, health care methods in adulthood, providing health knowledge, and sharing information.

*I wish there was more emphasis on stress management. As our children grow up, they sometimes tend to experience feelings of depression. So, I think that aspect is also essential (P14).*

Second, it was suggested that health self-advocacy programs be implemented as regular classes for short or long periods and that generalization and maintenance effects regarding the activation of the health self-advocacy program be investigated. Moreover, considering the importance of health or health care education, opinions have been raised regarding the necessity of early initiation of health care education and continuous guidance until adulthood.

*I loved this program so much that it would be significant if this kind of project became regular or even integrated into the school curriculum (P1).*

Finally, strategies to enhance the capacity of teachers and parents for health self-advocacy instruction are suggested. Training sessions for teachers to strengthen their ability to teach health self-advocacy were proposed, and the need to distribute program-related materials was discussed. Additionally, promoting parental involvement and support based on school-home connections and providing parent-focused education at their own level are indicated.

*It would be great to have learning materials like these available to me if I engaged in this kind of teaching in the future (T1).*

## 4. Discussion

### 4.1. Implications to reduce the stigma surrounding people with IDD

In this study, we explored the perceptions and experiences of students, parents, and teachers who participated in a health self-advocacy program with a school-home connection. Based on these findings, we discuss the implications of reducing the stigma surrounding people with IDD in health care issues.

First, by participating in the health self-advocacy program, positive changes were observed in students with IDD, which led to reducing the existing low expectations and stigma surrounding them and confirmed the potential to enhance health self-advocacy in individuals with IDD. The students showed changes in their perceptions of health self-advocacy and demonstrated changes in behaviors regarding health care across different health care domains. Furthermore, these changes were sustained, indicating that the learning experiences of the program were generalized. Additionally, teachers and parents who supported the students in the program gained a broader understanding of the current level of students with IDD. They also experienced a change in their perceptions of health self-advocacy. They also recognized the need to teach health self-advocacy and expressed their willingness to provide such support.

The positive changes observed in students with IDD and those around them through participation in the health self-advocacy program indicate the potential for breaking down the negative images and stigma associated with these individuals. People with intellectual disability are often undervalued, leading to stigma or misconceptions such as being perceived as “childlike” or having no potential for change or independence (20–22). An analysis of the image of individuals with disabilities in South Korea reported in newspaper articles during the past 30 years (1990–2020), published in April, including the Day of Persons with Disabilities, revealed that the image of individuals with disabilities as “incompetent and dependent” was the most prevalent (23). Therefore, the positive changes observed in students with IDD through the health self-advocacy program can contribute to reducing stigma toward them and addressing inequalities in health education and medical access.

Second, the positive changes in students with IDD and those around them indicate the necessity for professor support and expanded opportunities to enhance health self-advocacy in individuals with IDD. In this study, students were allowed to actively participate in setting goals in each health care domain and planning to reach those goals, along with basic and advanced sessions related to health care. They were also encouraged to take consistent actions at home, just as they do at school, through home guidelines and self-checklists. At school, teachers participated alongside students during the program, assisted in their participation, and supported the monitoring of behaviors for each health care goal. Professional support and personal assistance resulted in positive changes in the health care behaviors of students with IDD. Therefore, efforts should be made to provide appropriate professorial support for the health self-advocacy of students with IDD.

Third, it is possible to improve the outcomes of the transition to adulthood and the overall quality of life, by reducing the stigma surrounding individuals with IDD and inequalities in health care issues. Especially concerning transition outcomes, it is essential to focus not only on traditional quantitative measures (e.g., employment rates, deinstitutionalization rates, and post-secondary education enrollment rates) but also on the quality of transition outcomes related to well-being and quality of life. According to the Quality of Life Scale for Korean Adults with Developmental Disabilities (QLS-KADD), personal well-being includes both emotional and physical well-being (53). Considering that emotional and physical well-being carry significant weight to the overall quality of life, there is a need to expand educational opportunities related to these aspects.

### 4.2. Implications for future research and practice to enhance health self-advocacy

Considering the practical support needs proposed in the findings, the implications for enhancing the health self-advocacy of individuals with IDD are as follows:

First, conducting further research to develop and apply intervention programs for health self-advocacy is critical. The participants emphasized the importance of health care but found consistent and steady practices challenging. They expressed a desire for diverse intervention programs that could be implemented in various settings, including regular class time, over the long term. Previous research has repeatedly pointed out the lack of intervention programs to enhance the health self-advocacy of individuals with IDD (37, 46). Therefore, there is a need to develop health intervention programs specifically targeting individuals with IDD in various settings and age groups, such as health-focused integrated school clubs for students with and without IDD and health-related courses as elective subjects in high school credit systems. In particular, given that the participants reported that peers were a source of motivation and encouragement for students with IDD, a peer mentoring program for health care could prove useful by educating peers without disabilities about the unique health care needs of students with IDD and providing them with opportunities to work, through a health program, with adolescents with IDD. Additionally, health-related classes could be integrated into regular subjects such as practical arts and health education. The content of the current program can be further developed to create health programs for adults with IDD.

Second, it is necessary to distribute materials and provide education for teachers and parents, in addition to students with IDD. In this study, teachers expressed a desire for opportunities to enhance their expertise through professional development training and hoped for booklet-style materials that they could utilize. Similarly, parents wanted accessible education to adequately support their children’s health and self-advocacy. Given that both formal and informal support provided by teachers and parents had a positive impact on the self-directed health care and decision-making of students with IDD, offering training formats such as workshops, coaching, or consulting online and offline for teachers and parents could positively influence the health self-advocacy of students with IDD. Developing easily applicable workbooks or e-book formats, which teachers and parents can readily use as instructional materials, is also needed.

Finally, it is necessary to formulate policies that include individuals with IDD in health care and education for those without disabilities. Both the student and parent participants in this study frequently expressed difficulties in managing their mental health, emphasizing the need for support. However, the mental health challenges faced by individuals with IDD are often overlooked solely due to cognitive limitations, complex diagnoses, and difficulties in recognition (54, 55). Considering that individuals with IDD are at a higher risk of coexisting mental health issues than those without disabilities (56), appropriate support for mental health management is crucial. Unfortunately, many school-based mental health support systems lack specific provisions for students with IDD, and cases of reluctance to address their needs have been reported. The issue of health care disparities among individuals with disabilities, including access to mental health services, has been consistently highlighted in previous research (11, 14). Hence, policy-level support must ensure that individuals with IDD are not excluded from existing health support systems and that services are provided to meet their specific needs throughout their lives.

### 4.3. Limitations

The following limitations should be considered when interpreting the results of this study: First, while students and teachers participating in the health self-advocacy program with a school-home connection were interviewed, only six parents volunteered. Given the small number of participants, caution should be exercised when generalizing the results of this study. Second, although interviews were conducted with students with IDD who participated in this study, member checks of participants’ responses were only conducted with teachers and parents. Although teachers and parents were asked to review the students’ quotes, there was a limitation in verifying whether all the teachers and parents fully understood and validated the students’ words and intentions. Nevertheless, despite these limitations, this study is meaningful in confirming the potential for reducing the stigma surrounding students with IDD, enhancing health self-advocacy, and emphasizing the importance of instructional support and the potential of students with IDD.

## Data availability statement

The datasets presented in this article are not readily available because the individual datasets generated during the study are not publicly available owing to concerns about potential violations of participants’ privacy.

## Ethics statement

The studies involving human participants were reviewed and approved by the Review Board of Ewha Woman’s University (ewha-202204-0030-01). The participants provided their written informed consent to participate in this study.

## Author contributions

S-HL: Conceptualization, Data curation, Formal analysis, Funding acquisition, Investigation, Methodology, Supervision, Visualization, Writing – original draft, Writing – review & editing. H-NK: Data curation, Formal analysis, Investigation, Methodology, Writing – original draft. SK: Data curation, Formal Analysis, Investigation, Methodology, Writing – original draft.

## Footnotes

^1^https://clovanote.naver.com/
